# Secondary Metabolites, Ferulic Acid and *p*-Hydroxybenzoic Acid Induced Toxic Effects on Photosynthetic Process in *Rumex acetosa* L.

**DOI:** 10.3390/biom11020233

**Published:** 2021-02-05

**Authors:** M. Iftikhar Hussain, Manuel J. Reigosa

**Affiliations:** 1 Department of Plant Biology and Soil Science, Faculty of Biology, University of Vigo, Campus Lagoas-Marcosende, 36310 Vigo, Spain; mreigosa@uvigo.es; 2 CITACA, Agri-Food Research and Transfer Cluster, Campus da Auga, University of Vigo, 32004 Ourense, Spain

**Keywords:** phenolic compounds, physiological growth, common sorrel, natural herbicide, phytotoxicity

## Abstract

The elimination of broadleaf weeds from agricultural fields has become an urgent task in plant and environment protection. Allelopathic control is considered a potential approach because of its exclusive and ecological safety measures. Plant secondary metabolites also called allelochemicals are released from plant leaves, roots, stem, bark, flowers and play significant roles in soil rhizosphere signaling, chemical ecology, and plant defense. The present study was carried out to evaluate the impact of two allelochemicals; ferulic acid (FA) and *p*-hydroxybenzoic acid (pHBA) on photosynthetic characteristics; F_v_/F_m_: efficiency of photosystem II photochemistry in the dark-adapted state; ΦPSII: photosynthetic quantum yield; NPQ, non-photochemical quenching; qP, photochemical quenching, and photon energy dissipation (1−qP)/NPQ in *Rumex acetosa* following 6 days exposure. *R. acetosa* seedlings were grown in perlite culture, irrigated with Hoagland solution and treated with allelopathic compounds FA and pHBA and were evaluated against the photosynthetic attributes. Both compounds behaved as potent inhibitors of photosynthetic traits such as F_v_/F_m_, ΦPSII, qP, and NPQ in *R. acetosa*. Photon energy dissipation (1−qP)/NPQ increased significantly from days 3 to 6. Higher dissipation of absorbed energy indicates the inactivation state of reaction centers and their inability to effectively use the absorbed energy in photosynthesis. These results indicated the potential allelopathic application of FA and pHBA for control of broadleaf weed, *Rumex acetosa*.

## 1. Introduction

Weeds are noneconomic, unwanted and harmful vegetations that compete with other plants for water, space, nutrients, and light and thus cause significant loss to both agriculture and horticulture sectors worldwide [1]. Herbicides are an important source of weed control agents but due to their ecological impact and weed resistance problems have caused a significant pressure to search for plant based natural solutions as alternative weed management strategies. Natural products from living organisms, plants, fungi, and bacteria are a huge source of environmentally friendly “bioherbicides” that can overcome the herbicide resistance problem [2]. Plant based natural compounds have received much attention from scientists involved in natural product research [3]. Knowing the role of allelochemicals in plant interactions and distinguishing their effects from those of resource competition is controversial in plant interference investigations. Significant progress has been achieved during the previous year by the Callaway group [4] who reported that allelopathy is a separate phenomenon from general resource competition. Allelopathic compounds are present in almost all plant parts (like roots, seeds, leaves, fruits, and stems) and released to the environment through volatilization, leaching, exudation, or decomposition [5,6,7]. These toxic metabolites can be stored in the vacuole, polymerized or directly liberated, and finally released to the environment where they can act as allelopathic agents in the metabolism of neighboring plants, an action that is advantageous to the producer [7]. The intensity of the allelopathic effect in the field will depend, amongst others, on the different transformations that the organic compounds will suffer after release to the environment.

The chemical nature of allelochemicals is complex and diverse (organic acids, aldehydes, coumarins, quinones, flavonoids, alkaloids, and terpenoids, etc.), they largely derive from three principal biosynthetic routes; the shikimic acid route (benzoic and cinnamic acids and their derivatives such as coumarins, glycosides and alkaloids, etc.), and the acetic and mevalonic acid routes (terpenoids, steroids and complex quinones, etc.) [7,8]. Many natural products have been examined for phytotoxicity but the mode of action of the majority of known phytotoxins has not been elucidated [9]. Several reviews summarized the molecular target sites of natural phytotoxins with potential use as herbicides, mainly involving photosynthetic energy transfer, photosynthetic pigment synthesis, respiration, amino acid and protein synthesis, nucleic acid synthesis, lipid synthesis, membrane functions and lipid stability, gene expression and regulation, hormonal regulation, and disruption of the cell macrostructure and cell cycle [9,10,11]. However, available information indicates that there may be no preferred target sites for natural phytotoxins. Therefore, it is important to fully understand the action mode of phytotoxins, which can serve as the models or templates for new herbicides, as well as lead to the production of new tools for probing plant physiological and biochemical processes.

Allelopathic phenomena are usually different from pesticide effects as synthetic molecules are more specific, stronger, and have better defined effects in target species metabolism [11,12]. There are several thousands of natural products with allelopathic potential. However, present research was focused on ferulic acid (FA) and *p*-hydroxybenzoic acid (pHBA), both phenolic compounds that have been reported to possess phytotoxic potential [13]. Ferulic acid inhibited germination and seedling growth of several species including weeds [14,15]. Ferulic acid decreased the leaf water contents, expansion and root elongation, photosynthesis, and inhibited nutrient uptake [15]. The *p*-hydroxybenzoic acid (pHBA) is a widespread phenolic acid released into soil by root exudates, leaf leachates and decomposed tissues of different plants [16]. The phytotoxic properties of different allelochemicals including FA and pHBA have been recently documented by several authors [14,15,16,17]. However, their mode of action, interference with photosynthetic electron transport and photosystem II photochemistry, and the mechanistic details still remain undeciphered. Natural phytotoxins represent a diverse array of chemical structures that are unlikely to be designed in herbicide discovery efforts based on synthetic chemistry. 

Chlorophyll fluorescence is an important noninvasive ecophysiological technique that has been used by several researchers to elucidate the functional traits of plants and provide vital information about the plant–environment interaction [18,19]. Many scientific workers stated that thylakoid electron transport (light reaction), stomatal control of CO_2_ supply, and the carbon reduction cycle (dark reaction) were all affected by allelochemicals [16,17,18,19,20]. However, mechanisms related to the mode of action, interference with energy dissipation traits, photosynthetic electron transport, and photosystem II photochemistry still remain undeciphered [11]. 

Searching the mode of action is a complex phenomenon because there is not generic allelochemical behavior, sufficient allelochemical concentration is necessary for the studies, allelochemicals show new and several physiological and biochemical target sites, and multiple stresses can interact also on plant metabolism in synergistic or antagonistic ways. Previously, we also studied the phytotoxicity of various allelopathic compounds on photosystem II photochemistry, photosynthetic efficiency, quenching coefficients, and photon energy dissipation in various crops and weeds [6,10,12,18,21,22]. Physiological impact of allelochemicals and their primary target action sites within the plant photosynthetic apparatus and their interference with photosynthetic yield, chlorophyll fluorescence quenching, and photon energy dissipation in broad leaf weeds remains poorly understood. Therefore, *Rumex acetosa* was chosen as a model broadleaf weed to evaluate the toxicity of two potent phenolic compounds (FA and pHBA) on its photosynthetic apparatus. The specific objectives of this study include whether FA and pHBA are involved in the interference of chlorophyll fluorescence attributes, PSII functions and photon energy dissipation. Furthermore, insight on the biological activity of FA and pHBA and their interference with photosynthetic machinery and chlorophyll fluorescence quenching attributes in *R. acetosa* may also be ascertained. 

## 2. Materials and Methods

### 2.1. Phytotoxic Bioassays

Broadleaf weed, *Rumex acetosa* (cv. Belleville) seeds were surface sterilized and sown in plastic trays (32 × 20 × 6 cm) filled with a 5 cm deep layer of perlite (500 g/tray). The trays with seeds were placed in a growth chamber for 1 month. The plants were exposed to day/night temperatures of 28/20 °C, respectively, 09/15 h (light/dark) photoperiods, relative humidity (80%), and photosynthetic photon flux density (PPFD) at 275 μmol m^−2^ s^−1^. Following germination, seedlings were irrigated with 1:1 Hoagland nutrient solution (500 mL/tray) twice a week. Following 1 month growth period, seedlings were shifted to pots (10 cm) containing perlite (70 g) and supplied with 100 mL/pot Hoagland solution. Perlite is a typical growth substrate that provides sufficient plant support while totally avoiding microbial degradation of allelochemicals. To test the effects of phenolic compounds [(ferulic acid (FA) and *p*-hydroxybenzoic acid (pHBA)] (Sigma Chemical Company (St Louis, MO, USA)) on photosynthesis and photosystem II photochemistry attributes, *R. acetosa* seedlings were irrigated with either 1.5, 1.0, 0.5, 0.1 mM FA or pHBA or distilled water (containing the same concentration of ethanol as the controls). Phenolic acids were dissolved in a known volume of ethanol to make a stock solution of 3 mM. Distilled water was added to make different dilutions as reported above. To examine the time-course changes in photosynthesis, *R. acetosa* seedlings (growing in the perlite) were irrigated with either distilled water (control) or treatment solution (1.5, 1.0, 0.5, 0.1 mM) of either FA or pHBA. The allelochemicals solutions (FA or pHBA) and distilled water was applied at the rate of 100 mL/pot on alternate days (1, 3, and 5) and chlorophyll fluorescence measurements were carried out on each day up to 6 days. Three replicates of each treatment were repeated in a RCBD fashion. The photosynthesis was measured in terms of chlorophyll fluorescence traits on each day. 

### 2.2. Chlorophyll a Fluorescence Measurements 

Pulse-modulated chlorophyll *a* fluorescence monitoring system (FMS-2, Hansatech Instruments Ltd., Norfolk, UK) was used to measure the chlorophyll fluorescence traits and photon energy dissipation [18,22]. Chlorophyll fluorescence parameters of broadleaf weed *Rumex acetosa* leaves followed the method of Genty et al. [23]. The leaves were dark-adapted for 20 min using Walz leaf clips and then illuminated by saturation pulse light (1800 µmol m^−2^ s^−1^) for 3 s. The minimal fluorescence level in the dark-adapted state (F_0_) was measured using a modulate pulse (PPFD < 0.05 μmol (photon) m^−2^ s^−1^) too small to induce significant physiological changes. Maximal fluorescence (F_m_) was measured after applying a saturating actinic light pulse of 15,000 μmol (photon) m^−2^ s^−1^ for 0.7 s and the value of F_m’_ was recorded. The FMS-2 automatically recorded the photosynthetic attributes in terms of chlorophyll fluorescence quenching and nonquenching characteristics, including actual and maximum photosynthetic efficiency in the dark and light state. Chlorophyll fluorescence quenching and non-photochemical quenching were calculated as demonstrated by Kramer et al. [24], and Bilger et al. [25]. The excess photon energy of the PSII reaction center was calculated as (1−qP)/NPQ [18,22,26,27].

#### Calculations of the Electron Transport Rate

The electron transport rate (ETR) was calculated from the light-adapted fluorescence parameters as described by Liu [28]:

ETR = PAR *×* abs *×* β *×* ΦPSII

where ΦPSII represents the quantum yield of PSII; PAR is the incident photosynthetically active radiation; abs is the leaf absorbance. On average, a healthy leaf of higher plants absorbs ~84% of the incident PAR [29]. β represents the fraction of the absorbed photons that are subsequently absorbed by PSII. It is generally assumed that the absorbed photons are equally distributed between the two photosystems, PSII and PSI, and a value of β = 0.5 was used. 

Therefore, equation for the ETR was calculated as:

ETR = PAR *×* 0.84 *×* 0.5 *×* ΦPSII

The activity of water-splitting complex on the donor site of the PSII (F_v_/F_o_) was calculated as reported by Kalaji et al. [30] and Hassannejad et al. [31].

### 2.3. Statistical Analysis

The chlorophyll fluorescence traits data was analyzed by one-way ANOVA. Differences between concentration and treatments means were compared by the Dunnett test at the 0.05 probability level (when variance was homogeneous) or the Kruskal–Wallis test (when heterogeneous). All procedures were carried out in SPSS Version 19.0 (SPSS Inc., Chicago, IL, USA).

## 3. Results

### 3.1. Effect of Allelochemicals on Photosynthetic Attributes and Photosystem II Photochemistry

**Figure 1:**
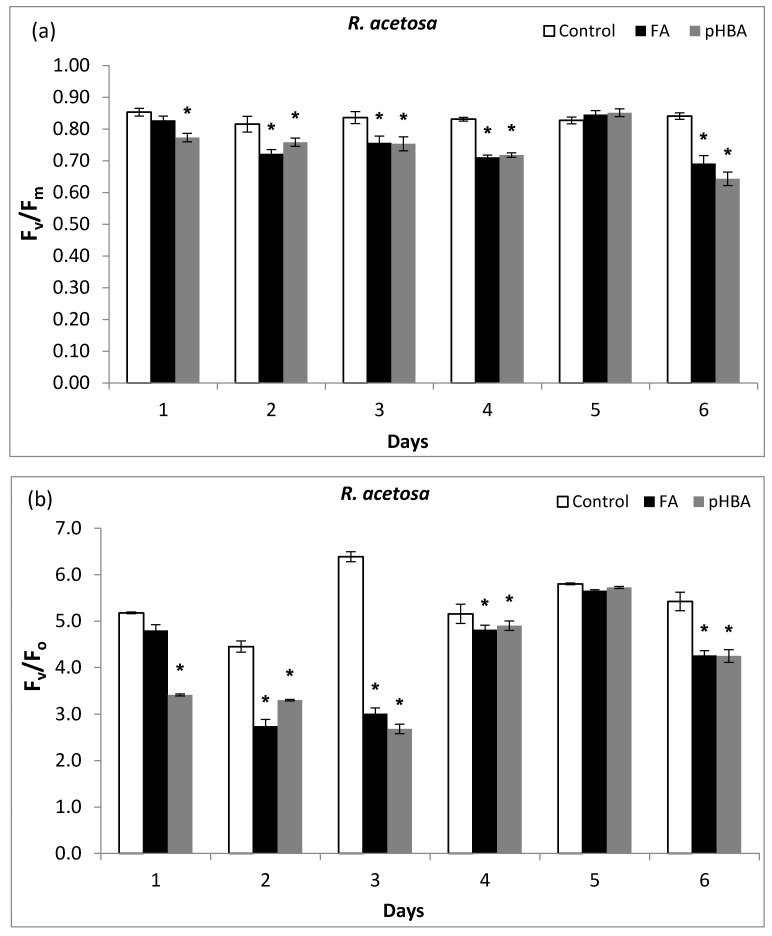
Changes in quantum efficiency of photosystem II PSII in the dark-adapted state (F_v_/F_m_) (**a**), and water-splitting complex values on the donor side of PSII (F_v_/F_o_) (**b**) in leaves of *Rumex acetosa* from days 1 to 6 following exposure to ferulic acid (FA) and *p*-hydroxybenzoic acid at 1.5 mM concentration. Every column in each graph represents the mean (± S.E.) of three replicates. * Asterisks indicate significant differences at *p* ≤ 0.05 with respect to control.

Chlorophyll *a* fluorescence (hereafter chlorophyll fluorescence) is a very sensitive indicator of stress effect on photosynthesis. The present study was carried out to evaluate the impact of two allelochemicals, ferulic acid (FA) and *p*-hydroxybenzoic acid (pHBA), on photosynthetic characteristics at four concentrations (1.5, 1.0, 0.5, 0.1 mM). However, to quickly confirm whether FA and pHBA affect photosynthetic activity through chlorophyll fluorescence attributes, here, we just present the results of highest concentration tested (1.5 mM) as compared with control. The F_v_/F_m_ is an important physiological index that indicates the maximum efficiency of photosystem II photochemistry and photon light energy absorbed and utilized in the PSII reaction centers. In dark-adapted broad leaves of *R. acetosa*, efficiency of photosystem II photochemistry (F_v_/F_m_) was significantly decreased following exposure to ferulic acid (FA) and *p*-hydroxybenzoic acid (pHBA) during 6 days (Figure 1a). The F_v_/F_m_ of *R. acetosa* was also affected by the allelochemicals (FA, pHBA) (Figure 1a) and gradually decreased following allelochemicals exposure. The F_v_/F_m_ leveled off in the control, while significant decrease was found at days 2, 3, 4, and 6 following exposure to both allelochemicals (Figure 1). In the control, F_v_/F_m_ values were 0.86. However, the F_v_/F_m_ decreased by16.27%, following treatment with FA at day 2 and by 17.85% after pHBA treatment on day 3 at 1.5 mM concentration as compared to control. At the 1.5 mM concentration, FA reduced the F_v_/F_m_ by 12.67% while pHBA inhibited the photosynthetic efficiency by 16.89% at day 3, respectively (Figure 1a). A remarkable reduction in *R. acetosa* was observed in F_v_/F_m_ values at day 6 when there was 17.83% and 23.53% reduction after FA and pHBA. Efficiency of the water-splitting complex on the donor side of PSII (F_v_/F_o_) remarkably decreased about 40% during days 2 and 3 at 1.5 mM concentration of pHBA compared with the control. More fatal effects were observed during days 2 and 3 followed by day 6 of the treatment with pHBA as compared to control (Figure 1b). The effect of FA was also lethal during days (2, 3, 6) when a significant reduction was observed in F_v_/F_o_ while more toxic impact was observed on day 2 when F_v_/F_o_ values decreased by more than 40% as compared to control (Figure 1b). The F_o_ values in *R. acetosa* seedlings grown under allelochemical stress after 6 days was higher in most of the days while F_o_ values were significantly decreased after 6 days treatment.

### 3.2. Phytotoxicity of Allelochemicals in Light Harvesting Complex (LHC II)

**Figure 2:**
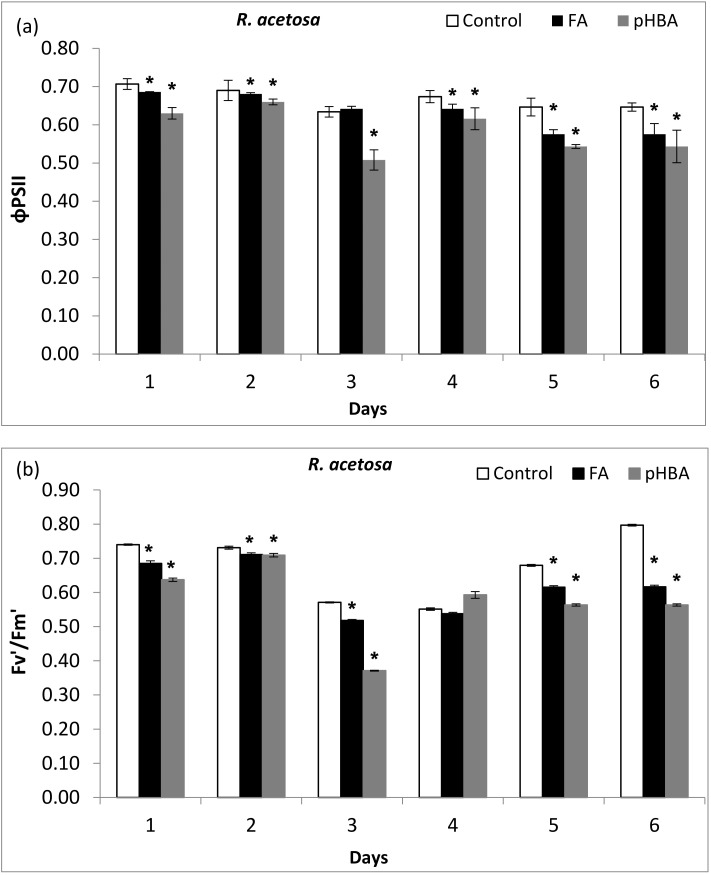
Changes in maximum quantum yield of photosystem II (ΦPSII) (**a**) and efficiency of PSII reaction centers in the light-adapted (F_v’_/F_m’_) (**b**) state in leaves of *Rumex acetosa* from days 1 to 6 following exposure to ferulic acid (FA) and *p*-hydroxybenzoic acid (pHBA) at 1.5 mM concentration. Every column in each graph represents mean (±S.E.) of three replicates. * Asterisks indicate significant differences at *p* ≤ 0.05 with respect to control.

ΦPSII is another valuable index that highlights the ratio between the yield of PSII photochemical quantum and total photochemical quantum. Higher concentration of FA and pHBA reduced the ΦPSII in *R. acetosa* during all days (Figure 2a). It showed that ΦPSII reduction was a coincidence with decrease in the efficiency of excitation energy trapping of PSII reaction centers. Moreover, inhibition in ΦPSII was more prominent after treatment with pHBA than FA. In comparison with the control, ΦPSII of *R. acetosa* significantly decreased by (19.04%) at day 3. The predominantly reduction in ΦPSII indicates that excitation energy trapping of PSII reaction centers was reduced (Figure 2a). There was significant inhibition in F_v’_/F_m’_ values during days 1, 2, 3, 5, and 6 after treatment of adult plants of *R. acetosa* with both allelochemicals (Figure 2b).

### 3.3. Chlorophyll Fluorescence Quenching Analysis and Tolerance Potential of LHC

**Figure 3:**
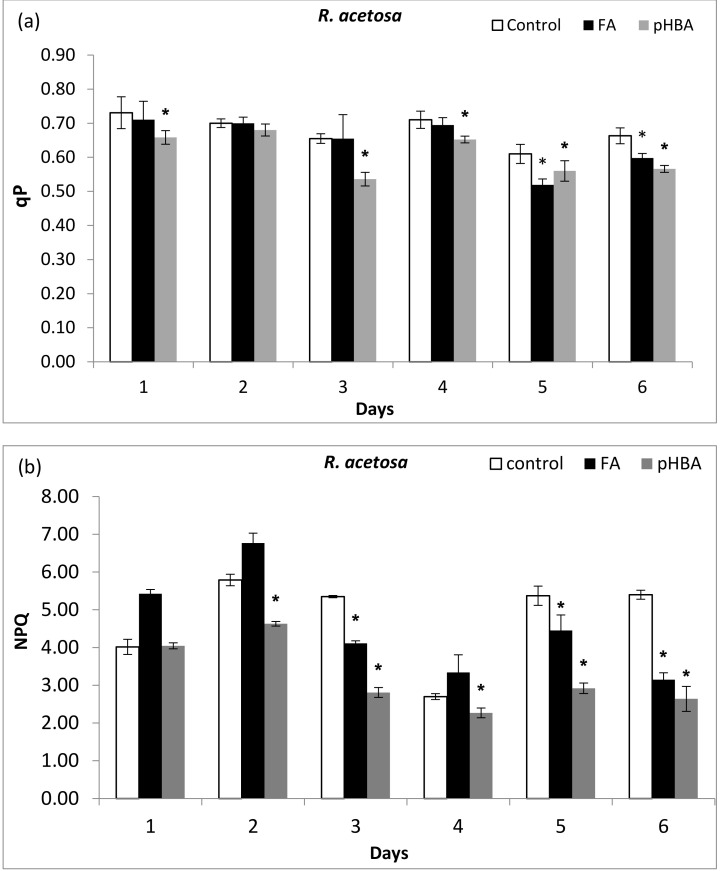
Changes in chlorophyll fluorescence quenching (qP) (**a**) and non-photochemical fluorescence quenching (NPQ) (**b**) in leaves of *Rumex acetosa* from days 1 to 6 following exposure to FA and pHBA. Every column in each graph represents the mean (± S.E.) of three replicates. * Asterisks indicate significant differences at *p* ≤ 0.05 with respect to control.

The photochemical quenching demonstrates the effective photochemical state of PSII photochemistry regarding the fraction of PSII centers that remain open or oxidized at any time. The impact of FA and pHBA was not consistent during different days. However, pHBA significantly decreased the qP values on days 1, 3, 4, 5, and 6, while FA reduced the qP values on day 5 and 6 (Figure 3a). The *R. acetosa* seedlings showed poor tolerance following FA and pHBA exposure and prominent effects were observed during days 5 and 6 after exposure to FA and pHBA (Figure 3a). The NPQ values were significantly decreased in *R. acetosa* and this reduction was more prominent on days 3, 4, 5, 6 (Figure 3b) after allelochemicals exposure. FA decreased the NPQ level of *R. acetosa* seedlings by 17% and 42% following exposure to 1.5 mM concentration. Non-photochemical fluorescence quenching values in *R. acetosa* were significantly reduced after treatment with both FA and pHBA at 1.5 mM concentration compared to control during days 3, 4, 5, 6 (Figure 3b).

### 3.4. Impact of Phenolic Acids on Photon Energy Dissipation (1 − qP)/NPQ in R. acetosa

**Figure 4:**
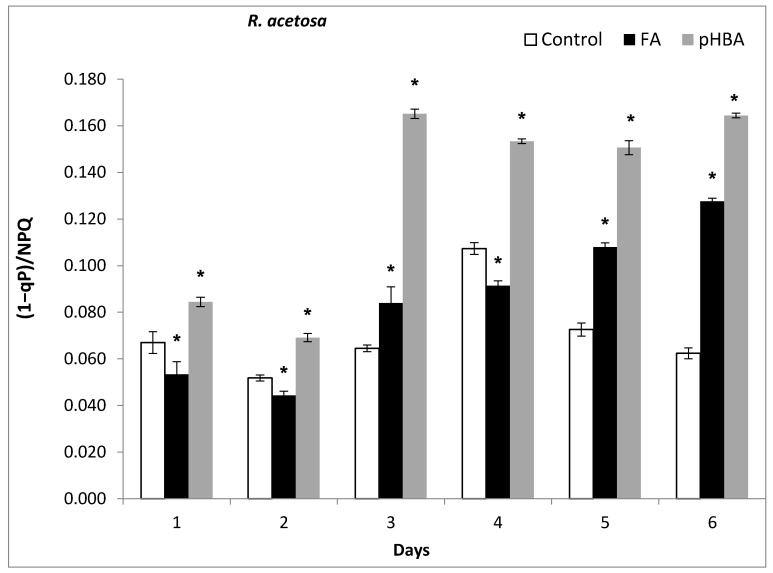
Changes in photon energy dissipated (1−qP)/NPQ) in leaves of *Rumex acetosa* from days 1–6 following exposure to FA, pHBA. Every bar in the graph represents the mean (± S.E.) of three replicates. * Asterisks indicate significant differences at *p* ≤ 0.05 with respect to control.

Different response pattern was observed in photon energy dissipation (1−qp)/NPQ in *R. acetosa* following 6 days exposure to FA, pHBA. During the initial 2 days, photon energy dissipation (1−qP)/NPQ was lower compared to control. However, it significantly increased and was significantly higher than control during days 3, 4, 5, and 6 (Figure 4). Higher dissipation of absorbed energy indicates the inactivation state of reaction centers and their inability to effectively use the absorbed energy in photosynthesis (Figure 4).

### 3.5. Photosynthetic Electron Transport (ETR) Responses under Allelochemical Stress

**Figure 5:**
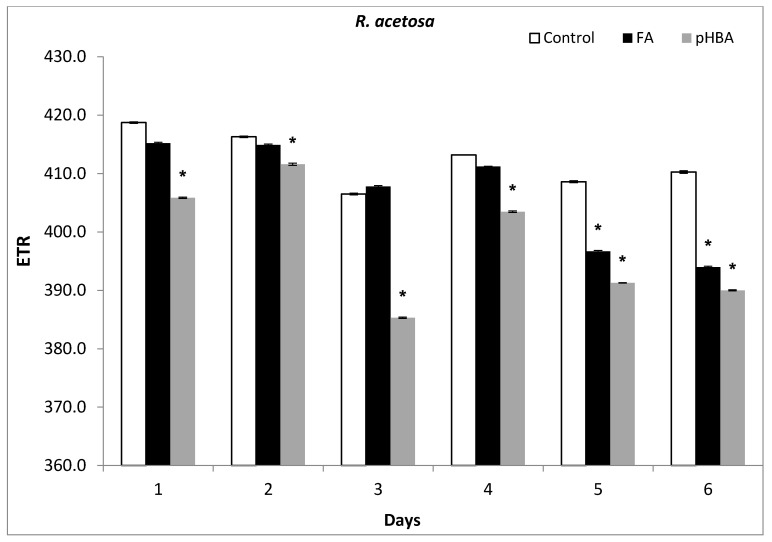
Changes in photosynthetic electron transport rate (ETR) in leaves of *Rumex acetosa* from days 1 to 6 following exposure to FA and pHBA. Every column in each graph represents the mean (±S.E.) of three replicates. * Asterisks indicate significant differences at *p* ≤ 0.05 with respect to control.

The photosynthetic electron transport (ETR) in *R. acetosa* was decreased by pHBA at days 1–6. The ETR values decreased by 19.9%, 8.5%, 15.9%, and 18.4% when *R. acetosa* seedlings were exposed to 1.5 mM pHBA during days 3, 4, 5, and 6 (Figure 5). Similar results were obtained after treatment with FA during days 4–6. The reduction of ETR was more prominent following exposure to pHBA than FA and showed more phytotoxicity than FA following 6-days treatment (Figure 5).

## 4. Discussion

Our results demonstrate the phytotoxicity of ferulic acid (FA) and *p*-hydroxybenzoic acid (pHBA) in the model broadleaf weed (*Rumex acetosa* L.), and explored their interactions with plant photosystem II photochemistry, fluorescence quenching coefficients, and photon energy dissipation attributes. First, FA and pHBA strongly inhibit PSII photochemistry (Figure 1 and Figure 2). The same mechanism has been reported by other phytochemicals including pHBA, FA and cinnamic acid in previous studies [20,21,22,23,32,33,34]. Ferulic acid and *p*-hydroxybenzoic acid are typical phenolic compounds, released by grasses, such as *Avena fatua*, *Triticum aestivum*, *Camelina alyssum*, *Imperata cylindrica*, *Sorghum bicolor,* and *Saccharum officinarum* [21,33], which are very often cultivated in crop rotation systems with other plants including maize in Mediterranean basin of Spain. There are different pathways for the release of allelochemicals into the soil rhizosphere such as plant litter leachates, tissue decomposition, exudates from the below ground parts (roots) and pHBA is typical example of this phenomena from *Triticum aestivum* L. and wild oat (*Avena fatua*) [35]. The phytotoxic potential of pHBA has been previously documented in soybean (root length inhibition) and wheat (coleoptile growth reduction) [33]. Until now, there has been little evidence to show that FA and pHBA are directly involved in photosynthesis inhibition in the broadleaf weeds, especially *R. acetosa*. In this paper, there was a significant retardation in photosystem II photochemistry, showing the greatest effects at 1.5 mM (Figure 1). Following 6 days treatment with FA or pHBA, the photosynthetic efficiency (F_v_/F_m_) in *R. acetosa* was significantly decreased and both allelochemicals caused a critical loss in photosynthesis. This worse effect was more prominent in plants treated with pHBA and appeared as soon as 6 h of treatment (Figure 1). Hussain and Reigosa [18] showed that continuous application of cinnamic acid and Benzoaxzolenine (1.5 mM) led to a decreased CO_2_ fixation rate, however, the observed reduction in photosystem II photochemistry rate was measured from days 1–6 after allelochemical treatment. F_v_/F_m_ decreased by 9–10%, and electron transport rate decreased by 20–25% in *Lolium perenne*, *Dactylis glomerata,* and *Rumex acetosa*, and the photosynthesis rate was significantly decreased and the main mechanism for this inhibition was suggested to be stomata closure.

The changes in CO_2_ assimilation may lead to stomatal closure. In this study, water-splitting complex on the donor side of PSII values (F_v_/F_o_) significantly decreased during all days. Severe impact was observed during days 2, 3, 4 followed by day 6 of the treatment with pHBA and FA as compared to control (Figure 1b). The effect of FA was also lethal during all the days except day 5 when a recovery in F_v_/F_o_ was observed, while more toxic impact was observed on day 3 when F_v_/F_o_ values decreased by 50% as compared to control (Figure 1b). The F_v_/F_o_ values and photochemical efficiency (F_v_/F_m_) was decreased after salt stress. The efficiency of the oxygen evolving complex and F_v_/F_o_ are very sensitive in the electron transport chain of photosynthesis. A sharp decline of this ratio results in the disruption of electron transport [36]. It was confirmed that the inhibition of electron transport by allelochemicals was mainly from the donor side of PSII-OEC. Similar results were documented by other researchers [31,36,37].

It is important to understand the biochemical and physiological traits of the target plant species that will ultimately help to investigate the action mode of allelochemicals. The much great diversity of identified allelopathic compounds in nature implies a high variability in their mode of action. Works found in literature show that allelochemicals interfere with several and diverse metabolic processes in plants [38,39,40]. Application of cinnamic acid (100 μM) caused significant reduction in photosynthetic rate but there was no effect on efficiency and quantum yield of ΦPSII [41]. However, when the concentration of cinnamic acid was increased to 250 μM, a drastic inhibition in the values of ΦPSII was obtained [42]. The higher F_o_ values obtained in *R. acetosa* seedlings following allelochemical treatment showed that the number of inactive reaction centers has increased and was responsible for interference in the process of electron transfers during the Q_A_ reduction cycle [18]. Similarly, other researchers also reported an inhibition on electron transport of thylakoids from *Spinacea oleracea* and *Amaranthus retroflexus* in presence of sorgoleone. They suggested that perhaps allelochemical compounds can block the electron acceptor Q_B_ (of the protein D_1_), avoiding the reoxidation of Q_A_- by Q_B_ and acting as herbicides known to inhibit the Photosystem II [43,44]. Another study highlighted that cinnamic acid also decreased all components of photosynthesis (F_v_, F_m_, F_v_/F_m_, and ΦPSII) in *Lactuca sativa*, following treatment to CA at 1.5 mM concentration [10].

Allelochemicals (FA, pHBA) application significantly inhibited the photosynthetic capacity of leaves, which was accompanied by a decrease in the ΦPSII. The simultaneous decrease in the ΦPSII during days 2, 5, and 6 caused by FA and pHBA treatment is attributed to (i) reduction in the initial RuBisCO activity and (ii) decrease in the activity of other Calvin-cycle enzymes that may result a decrease in the rate of RuBP regeneration (*J*_max_) [45]. The reduction in ΦPSII was primarily attributed to the unutilized ATP and NADPH during photosynthesis [18]. This will not be further compensated by photorespiration or water-water cycle. According to Baker [46] and Parizotto et al. [47], ΦPSII is linearly correlated with CO_2_ assimilation under non-photorespiratory conditions. The ΦPSII was significantly decreased following both allelochemical treatment (Figure 2a). This indicates non-stomatal limitation for the photosynthesis. Other scientists also documented similar phytotoxic effects of benzoxazolin-2-(3*H*)-one (BOA) on soybean [47]. The ΦPSII was also decreased in perennial rye grass and broadleaf weeds following allelochemicals stress [18,22].

Efficiency of photosystem II photochemistry in the light state (F_v’_/F_m’_) was highly decreased by FA and pHBA. Our results showed that both phenolic compounds caused significant damage in the photosynthetic machinery and CO_2_ assimilation and entry of CO_2_ into the leaves. Inhibition of the CO_2_ passage due to stomata closure will result in the production of reactive oxygen species, xanthophylls pigments degradation, lipids and proteins oxidation, and ultimately plant growth and development [48]. We observed different phytotoxic response of allelochemicals on the photon energy dissipation “(1−qP)/NPQ”. The photon energy dissipation (1−qP)/NPQ increased 1–2-fold more than control during days 3–6. Higher dissipation of absorbed energy indicates the inactivation state of reaction centers and their inability to effectively use the absorbed photon energy in photosynthesis. Our data show that FA and pHBA clearly disturbed and disorganized the structure and function of thylakoid membranes and photosynthesis process. This can lead to disequilibrium and proper functioning of electron transport rate, ATP and NADPH production [49]. Therefore, our hypothesis was further supported by evidence that tested allelochemicals have affected the photosystem II photochemistry. These results support the prediction that as there was significant reduction in photosystem II photochemistry and photosynthetic electron transport, that will lead to the production of reactive oxygen species. These disturbances in photosystem caused inactivation of reaction centers and thus photosynthetic machinery was not able to fully utilize the absorbed photon energy and a lot of absorbed photon energy and consequently, excess photon energy was dissipated as heat [50,51,52]. Our results are in agreement with the previous reports that showed the direct inhibition of photosystem II photochemistry components, ion uptake, and interruption of dark respiration, ATP synthesis and ROS-mediated allelopathic mechanisms [43].

## 5. Conclusions

In summary, we found that allelochemical (FA, pHBA) stress significantly inhibited the photosynthetic machinery in broadleaf weed *Rumex acetosa*. Both allelochemicals drastically reduced the F_v_/F_m_, ΦPSII in leaves of *Rumex acetosa*. The elevated concentrations (1.5 mM) of both phytochemicals retard the photosystem II photochemistry parameters. The NPQ and qP values in *R. acetosa* leaves were significantly lower after FA and pHBA treatment. Higher dissipation of absorbed energy indicates the inactivation state of reaction centers and their inability to effectively use the absorbed energy in photosynthesis. The present article highlights the possible mode of action and damage caused by two potent allelochemicals to photosynthesis attributes using the chlorophyll fluorescence to study the FA and pHBA on photosynthetic machinery. The research and development of natural products is extremely important to understand their unknown chemistry and their physiological action mode to enhance our knowledge about their possible target sites and to use them as bioherbicides for weed control programs and to find out lead compounds for herbicide resistance weeds.

## Data Availability

The data presented in this study are available on request from the corresponding author.
